# Characterization of the Rhizosphere Bacterial Microbiome and Coffee Bean Fermentation in the Castillo-Tambo and Bourbon Varieties in the Popayán-Colombia Plateau

**DOI:** 10.1186/s12870-023-04182-2

**Published:** 2023-04-25

**Authors:** Andrés Felipe Solis Pino, Zuly Yuliana Delgado Espinosa, Efren Venancio Ramos Cabrera

**Affiliations:** 1 Corporación Universitaria Comfacauca - Unicomfacauca, Cl. 4 N. 8-30, Popayán, Cauca, 190001 Colombia; 2 Universidad Nacional Abierta y a Distancia - UNAD, Calle 5 # 46N -67, Popayán, Cauca, 190001 Colombia

**Keywords:** Rhizosphere microbiome, Coffee quality, Soil, Microorganisms

## Abstract

**Background:**

The microbial biodiversity and the role of microorganisms in the fermentation of washed coffee in Colombia were investigated using the Bourbon and Castillo coffee varieties. DNA sequencing was used to evaluate the soil microbial biota and their contribution to fermentation. The potential benefits of these microorganisms were analyzed, including increased productivity and the need to understand the rhizospheric bacterial species to optimize these benefits.

**Methods:**

This study used coffee beans for DNA extraction and 16 S rRNA sequencing. The beans were pulped, samples were stored at 4ºC, and the fermentation process was at 19.5ºC and 24ºC. The fermented mucilage and root-soil samples were collected in duplicate at 0, 12, and 24 h. DNA was extracted from the samples at a concentration of 20 ng/µl per sample, and the data obtained were analyzed using the Mothur platform.

**Results:**

The study demonstrates that the coffee rhizosphere is a diverse ecosystem composed primarily of microorganisms that cannot be cultured in the laboratory. This suggests that the microbial community may vary depending on the coffee variety and play an essential role in fermentation and overall coffee quality.

**Conclusions:**

The study highlights the importance of understanding and optimizing the microbial diversity in coffee production, which could have implications for the sustainability and success of coffee production. DNA sequencing techniques can help characterize the structure of the soil microbial biota and evaluate its contribution to coffee fermentation. Finally, further research is needed to fully understand the biodiversity of coffee rhizospheric bacteria and their role.

## Key message

It observed the Bourbon and Castillo coffee varieties to have a distinct microbial profile, indicating the presence of a diverse assemblage of soil and phyllosphere microorganisms, including non-culturable bacterial species.

## Background

Coffee is a globally significant crop cultivated in over 50 countries and is the most widely consumed beverage worldwide [1]. The active properties of coffee [2] have been reported to reduce the risk of pathologies such as diabetes [3] and Parkinson’s disease [4]. It estimates consumption of coffee to be approximately 2.5 billion cups per day [5], and it stands as the fifth most traded product globally. In Colombia, coffee constitutes the primary export product [6]. Currently, two main species of coffee are cultivated: Coffea arabica, known as arabica coffee, which represents 75–80% of global production, and *Coffea canephora*, known as *robusta coffee*, which represents about 20–25% of global production and differs from arabica coffee in terms of flavor, caffeine content, and production conditions [7].

The soil is a dynamic and complex ecosystem that is essential for the growth and development of plants. For a coffee plant to produce 100 pounds of green coffee, it must extract approximately 1.45 kg of nitrogen, 0.28 kg of phosphorus, and 1.74 kg of potassium from the soil [8]. Soil fertility, defined as the ability of the earth to provide essential nutrients to plant roots, can sometimes be limited. However, microorganisms can help solubilize these nutrients and make them available to plants. These microorganisms are crucial in maintaining soil fertility and plant health [9]. The bacterial community of coffee soils is diverse and can be influenced by environmental conditions, coffee varieties, and processing methods, ultimately impacting the quality of coffee beans [10]. Besides maintaining soil fertility, soil bacteria perform essential ecosystem functions, such as nutrient cycling, organic matter decomposition, biological nitrogen fixation, and phosphorus solubilization [11]. As a perennial crop, coffee can harbor many beneficial microorganisms in its rhizosphere, including phosphate-solubilizing and nitrogen-fixing bacteria, which can significantly supply the plant’s nutritional needs [12]. However, the specific species of rhizosphere bacteria that provide these benefits need to be better understood. Further research is needed to reveal their incredible biodiversity [13, 14], identify the strains that can modulate rhizosphere microbial structures, and determine their contribution to coffee bean fermentation and the quality of the resulting beverage [15], similar to what has been reported in products such as wine [16] or tomato [17].

The study of rhizosphere bacteria is a challenging task because of the large number of organisms that exist in the soil. It is essential to characterize and identify these microorganisms to advance ecological studies of plant rhizospheres and coffee cultivation. Among the modern methods available, molecular sequencing is highly effective [18]. It involves a series of biochemical methods and techniques that permit the determination of the order of nucleotides in a DNA oligonucleotide [19], specifically, regions of the 16 S Ribosomal ribonucleic acid (rRNA) that can identify microorganisms present in the rhizosphere and fruit and explain their role in productive processes [13, 20].

Given the significant role of soil bacteria in plant health and coffee production, it is essential to recognize the impact of microbial structure and its function in the fermentation process of this crop. This research aims to identify the microbial composition of the rhizosphere of the Bourbon and Castillo coffee varieties and determine their contribution to the fermentation process of washed coffee from the Popayán-Cauca plateau in Colombia. Recognizing the microbiological correlation between soil, plant, and fermentation process can provide critical insights into the variables that influence the quality of the final product.

Finally, it organized the document as follows: Sect. 2 presents a review of the relevant literature on this topic. Section 3 describes the materials and methods used to collect and analyze the experimental data. Section 4 presents the study’s results, including descriptive and inferential statistical analyses. Finally, Sect. 5 summarizes the main findings of the research and the conclusions reached.

## Related work

The microbial diversity in soil plays a critical role in terrestrial ecosystems’ nutrient cycling and decomposition processes [21]. Microorganisms perform various biochemical processes in the soil, such as oxidation-reduction and interspecific and intraspecific interactions [22]. In particular, for crops like coffee, studying microbial diversity is crucial because microorganisms’ habitat and biochemical processes can contribute to benefits such as increased productivity and soil conservation, which are essential for the growth of products that rely on soil nutrients [23].

Several studies have investigated DNA sequencing techniques to understand coffee production. Silva et al. [24] examined the microbial biota associated with coffee’s dry and wet processing by taking soil samples from trees across different production cycles. Their sequencing analysis revealed that bacteria and filamentous fungi were the most commonly found organisms, but their appearances varied. They concluded that the microbial flora in coffee production is much more complex and varied during the wet stage than in the dry stage.

Velmourougane et al. in [25] evaluated the long-term impact of organic and conventional coffee cultivation methods using soil DNA sequencing. Their findings suggested that organic methods resulted in higher rates of macrofauna, microbial population, and diversity than the conventional system. This indicates that coffee soil cultivated under organic systems has better long-term properties than conventional ones. Another study [26] analyzed the influence of continuous cultivation on soil chemical properties and microbial communities using DNA sequencing. The sequencing results from soil samples indicated that long-term monoculture decreased soil pH and reduced soil bacterial and fungal richness.

Furthermore, Veloso et al. in [27] investigated how fermentation influences the final quality of coffee and the interactions between soil, fruit, altitude, and slope exposure on the microbiome of coffee plants using DNA sequencing. Their findings suggested that environmental factors contribute to the structure of bacterial and fungal communities and can influence the growth of these organisms.

Finally, despite the importance and utility of employing various methods and techniques to study the organisms present in the soil where coffee is grown, no studies have been found that investigate the relationship between the microbial flora of the coffee soil with the organoleptic properties and how these microorganisms influence the fermentation processes in the post-production of the fruit and the quality of the beverage, as far as the authors are aware. Therefore, there needs to be more knowledge on this research topic.

## Results

### Estimating the diversity and richness of the bacterial community in the rhizosphere and coffee bean fermentation

**Fig. 1:**
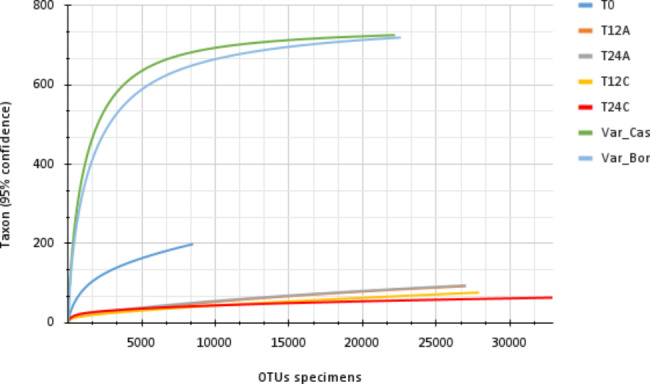
Rarefaction curves based on operational taxonomic units. It grouped taxa using a 95% confidence interval. Each curve represents the means of the biological replicates of the different coffee rhizosphere and bean fermentation samples

A total of 200,000 sequence reads of 16 S ribosomal RNA were obtained, and after filtering low-quality reads, 34,500 reads were kept for analysis. It compared the diversity among the different samples using rarefaction curves with 5% similarity, which showed that all samples except for the coffee fermentation time sample reached the saturation point (Fig. 1). It found the diversity of microorganisms in coffee fermentation to be low compared to the diverse microorganisms in the coffee rhizosphere.

**Fig. 2:**
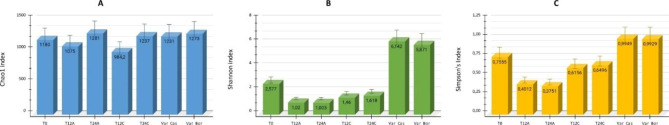
Effect of fermentation and coffee variety on the ecological indexes Shanon (**A**), Chao _1 (**B**), and Simpson’s (**C**.) in the bacterial structure. Letters indicate significant differences between treatments applied in the Tukey test (p > 0.05)

The Simpson 1-D, Shannon-Wiener, and Chao-1 diversity estimators were used to evaluate the bacterial community’s diversity and richness. Analysis of variance indicated the effect of fermentation time and temperature on bacterial community richness (Fig. 2A), except for T0. Samples from T-24-A, T-12-C, and T-24-C showed significantly lower richness values than T0. The ambient temperature at 12 h altered the richness, while at 24 h, this parameter decreased significantly. In contrast, samples of coffee beans fermented at warm temperatures showed decreased richness in the different samples.

Regarding the diversity parameter (H′) (Fig. 2B), sampling at T0 had the highest diversity compared to sampling at temperature and warmth. The ambient temperature indicated the lowest diversity, followed by warm temperature, indicating that the temperature variable modulates the diversity of the bacterial community in the coffee fermentation process. Sampling time did not affect diversity (12 and 24 h). Figure 2 C also showed that the ambient temperature trends at 12 and 24 h were like the previous graphs and were in line with the results shown by the diversity index.

### Characterization of the microbial communities

**Fig. 3:**
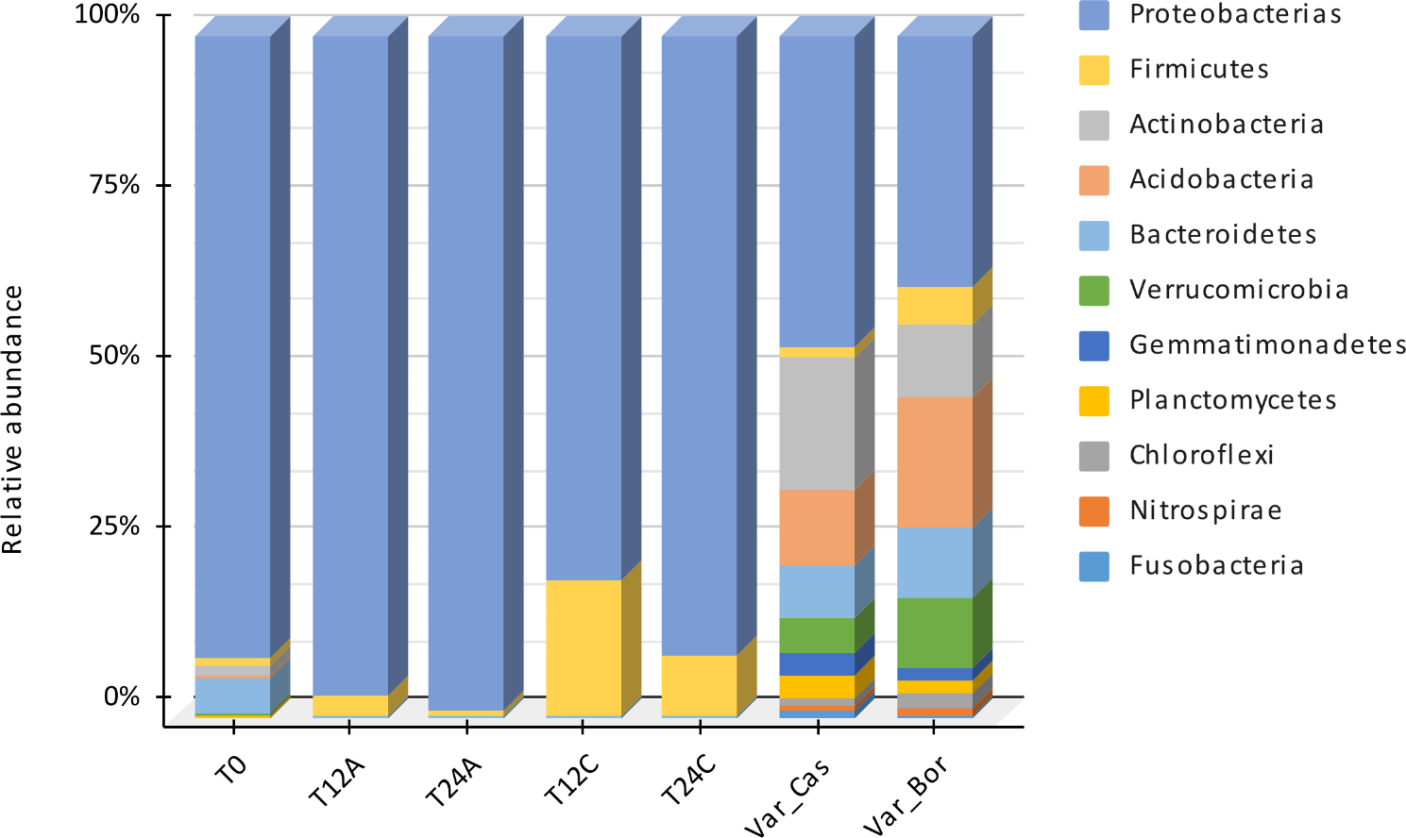
Distribution of the phyla of the bacterial community present in coffee fermentation. The nomenclatures T0 indicate zero fermentation time, T12A sampling at 12 h at room temperature (18–20 °C), T24A sampling at 24 h of fermentation at room temperature, T12C and T-24-C sampling at 12 and 24 h at hot temperature (24 °C) respectively

The Proteobacteria phylum was the most prevalent among the bacterial community across different sampling points and temperatures, accounting for 76.2% of the observed microbial composition. Other prominent phyla included *Firmicutes* (4.6%), *Bacteroidetes* (3.1%), and *Acidobacteria* (4.5%), while the remaining phyla did not exceed 3% (Fig. 3). Bacterial microbiome analysis during fermentation indicated changes in the microbial composition with increasing fermentation time and temperature, which favored the growth of Proteobacteria and Firmicutes phyla. It detected *Bacteroidetes* and *Actinobacteria* at the initial sampling point (T0) but disappeared with prolonged fermentation time and temperature. A greater diversity of phyla was observed in the rhizosphere, including *Proteobacteria*, *Actinobacteria*, *Acidobacteria*, *Bacteroidetes*, *Verrucomicrobia*, *Gemmatiomonadetes*, *Chloroflexi*, *Planctomycetes*, *Nitrospira*, and *Fusobacteria* groups, with the last six phyla being typical of rhizospheres.

The microbial composition of the rhizosphere varied between the Castillo-Tambo and Bourbon coffee varieties. Taxonomic analysis of the fermentation production process revealed the prevalence of *Enterobacteriales*, *Rhodospirillales*, and *Lactobacillales* orders. In contrast, in the rhizosphere, *Sphingomonadales*, *Sphingobacteriales*, *Rhizobiales*, *Burkholderiales*, *Actinomycetales*, *Verrucomicrobiales*, *Acidobacteriales*, *Solirubrobacteriales*, *Acidimicrobiales*, *Solibacterales*, *Gemmatimonadales*, *Nitrosomonadales*, *Desulfuromonadales*, *Ktedonobacteriales*, *Fusobacteriales*, *Syntrophobacterales*, and *Nitrospirales* orders were dominant.

**Fig. 4:**
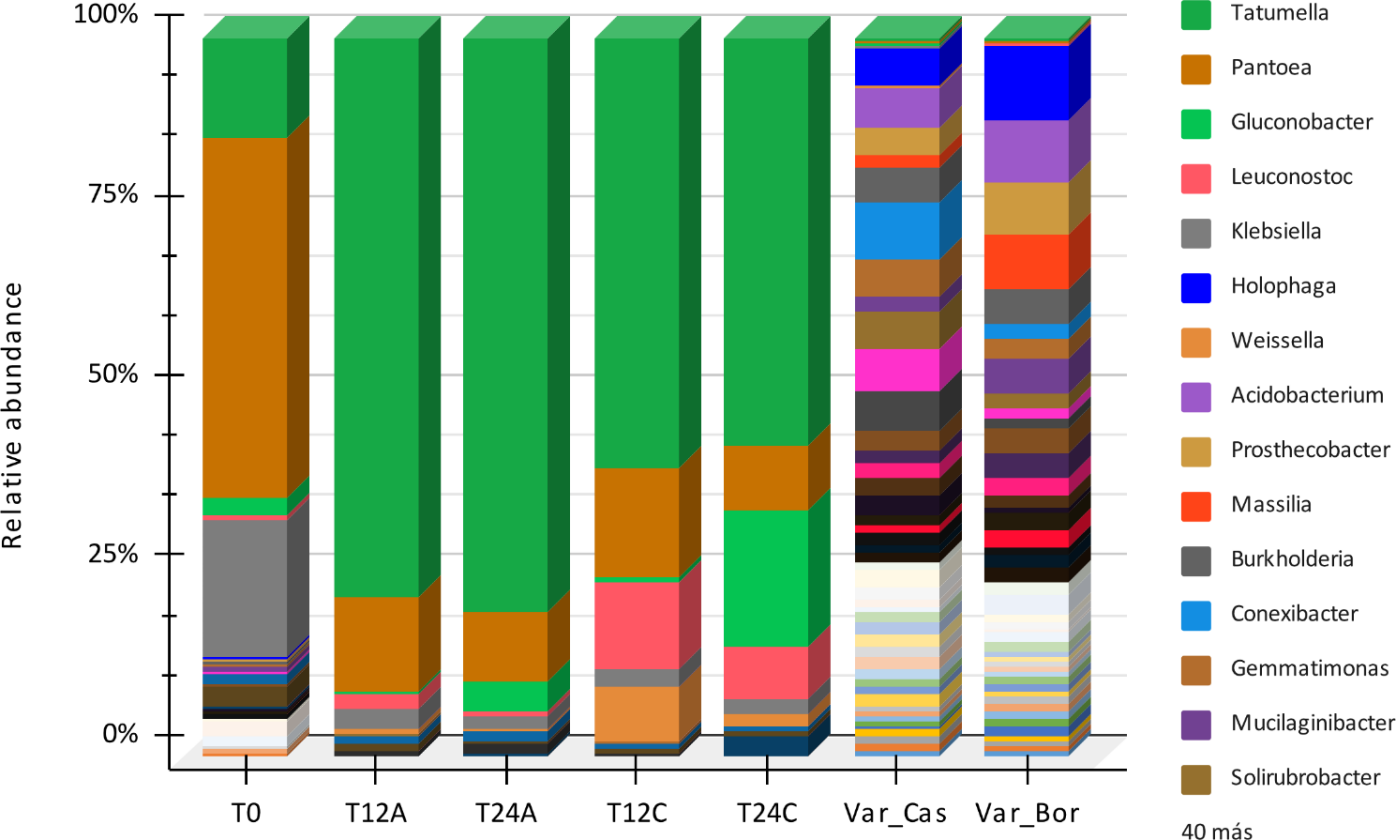
Genus distribution of the bacterial community is present in coffee fermentation and Bourbon and Castillo coffee varieties. The nomenclatures T0 indicate zero fermentation time, T12A sampling at 12 h at room temperature (18–20 °C), T24A sampling at 24 h of fermentation at room temperature, T12C and T-24-C sampling at 12 and 24 h at hot temperature (24 °C) respectively

In relation to the diversity of microorganisms present in the samples analyzed, several genera were identified (Fig. 4), such as *Verrucomicrobium*, *Tatumella*, *Planctomyces*, *Geobacter*, *Pantoea*, *Stella*, *Cupriavidus*, *Chitinophaga*, *Terrimonas*, *Klebsiella*, *Pelobacter*, *Nitrospira*, *Reyranella*, *Phenylobacterium*, *Hylemonella*, *Aciditerrimonas*, *Actinoallomurus*, *Streptomyces*, *Sphingomonas*, *Steroidobacter*, *Niastella*, *Thermoflavimicrobium*, *Nitrosovibrio*, *Holophaga*, *Bacillus*, *Pseudomonas*, *Koribacter*, *Arthrobacter*, *Rhizobium*, *Kaistobacter*, *Anaeromyxobacter*, *Erwinia*, *Pedosphaera*, *Candidatus solibacter*, *Shigella*, *Rhodoplanes*, *Bradyrhizobium*, *Solirubrobacter*, and *Mucilaginibacter*.

Specifically, at time T0, the highest abundances of the genera *Pantoea*, *Sphingomonas*, and *Tatumella* were observed. At time T12A, the most abundant genera were *Acinetobacter*, *Erwinia*, and *Tatumella*. At time T24A, the most abundant genera were *Tatumella*, *Shigella*, and *Pantoea*. At time T12C, the most abundant genera were *Tatumella*, *Pantoea*, and *Weissella*. At time T24C, the most abundant genera were *Tatumella*, *Gluconobacter*, and *Leuconostoc*.

The abundance of the different microbial genera varied between the different times. Some genera, such as *Tatumella* and *Pantoea* were consistently abundant across multiple time points [28]. Other genera, such as *Leuconostoc*, and *Gluconobacter* were more abundant at later times, suggesting that they may play a role in the later stages of coffee fermentation [29].

### Beta diversity pattern of the rhizosphere community and fermentation

**Fig. 5:**
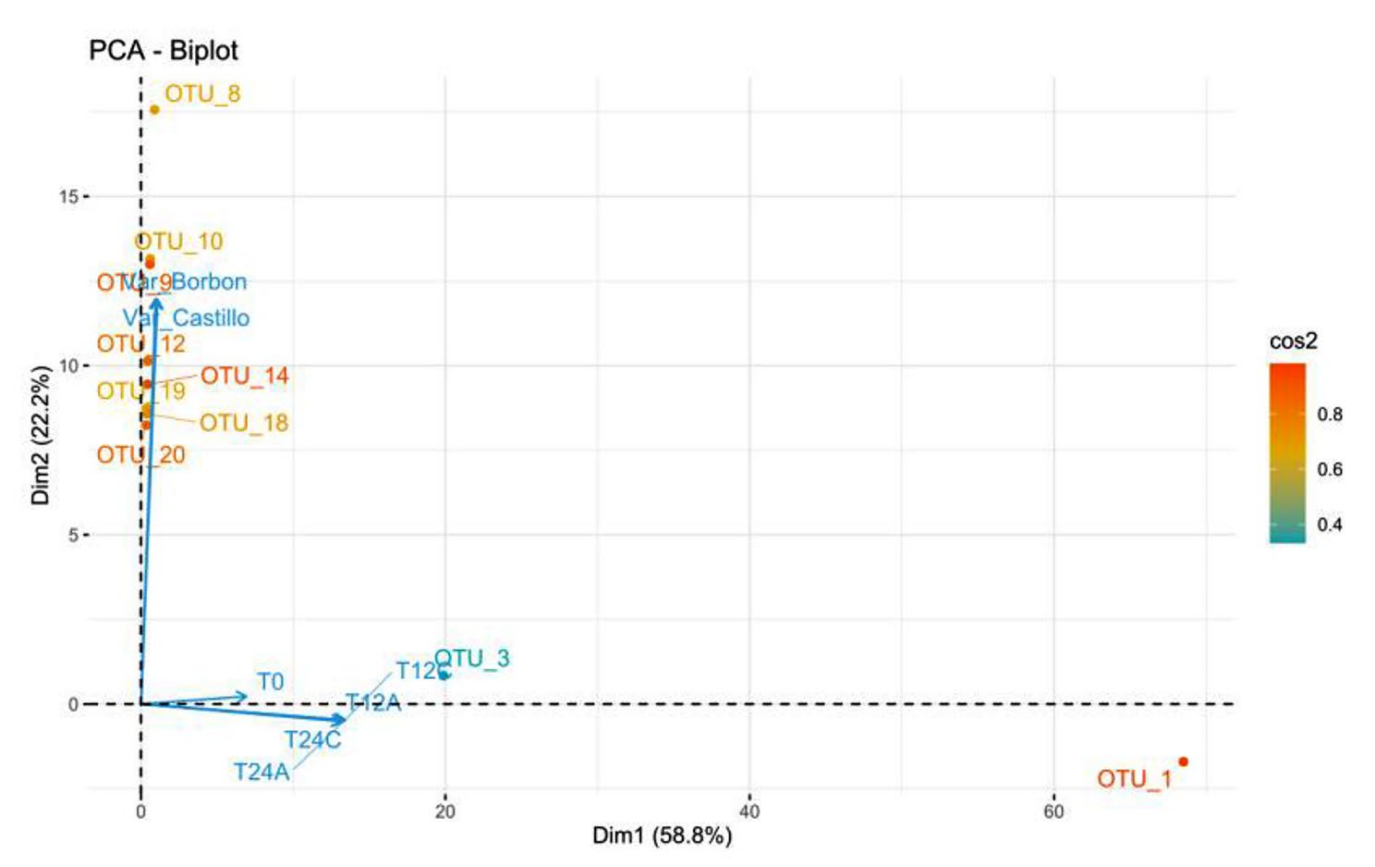
biplot graph relating genetic samples to organoleptic properties, where almost 80% of the total variability is explained

To explore beta diversity, a Principal Component Analysis (PCA) was conducted (Fig. 5) to examine the relationship between variables and Operational Taxonomic Units (OTUs). It selected only OTUs representing over ten sequences in this investigation, resulting in a dataset of 1265 OTUs and seven variables. Outlier analysis of the graphs did not reveal any anomalies. The first two dimensions of the PCA explained 81.06% of the total inertia of the dataset, suggesting that 81.06% of the overall variability of the OTU cloud is explicable in the plane. This high percentage indicates that the primary component explains a significant part of the dataset’s variability. The variability explained by this plane is considerably more significant than the reference value, which corresponds to 31.68%, highlighting the relevance of the variability captured by the plane (The reference value being 0.95 quantiles).

The PCA analysis confirmed that it separated the fermentation process samples from the rhizosphere samples in the Cartesian plane. Dimension one revealed that the fermentation samples oppose OTUs with a strongly positive coordinate on the axis to the right of the graph. The samples T24A, T12C, T12A, T24C, and T0, which share high values for the variables, confirmed this separation. Notably, the variables T12A, T24A, T12C, and T24C indicated a high correlation with this dimension (0.97, 0.96, 0.97, 0.94, respectively), indicating the microorganisms present in these samples are alike. In contrast, T0 displayed a lower correlation and formed a subgroup, indicating where the microorganisms differed from the fermenting samples. The second group, represented by the rhizosphere samples, was found in dimension two and faced individuals characterized by a strongly positive coordinate on the axis towards the top of the graph with the OTUs.

**Fig. 6:**
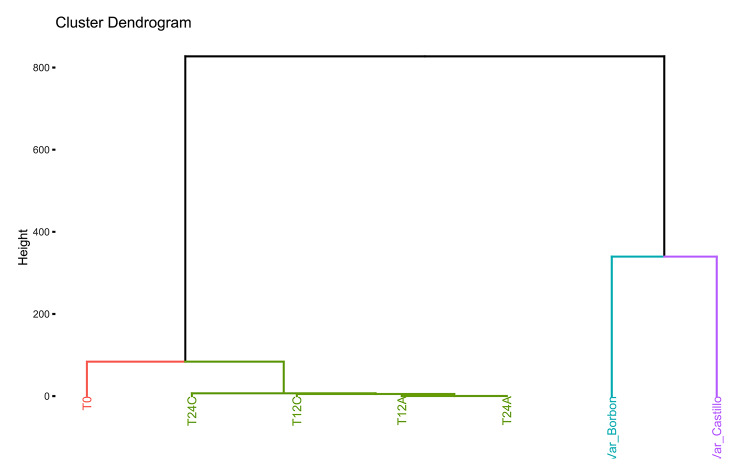
Dendrogram showing the relationship between the different fermentation temperatures and the Castillo and Bourbon coffee varieties

A graphical representation (Fig. 6) was constructed to classify individuals based on their unique sets of variables, identifying four distinct clusters. A set of variables characterizes each cluster, with the strength of each variable listed in descending order.

Cluster 1 is defined by high values for variables including OTU_2101, OTU_1, OTU_2183, OTU_1267, and OTU_925, while displaying low values for variables like OTU_491, OTU_685, OTU_243, OTU_113, OTU_175, OTU_290, OTU_991, OTU_581, OTU_990, and OTU_190. This cluster is composed of individuals such as T12C. In this line, cluster 2 is characterized by high values for variables such as OTU_819, OTU_715, OTU_524, OTU_1099, OTU_1079, OTU_1055, OTU_1031, OTU_643, OTU_404, and OTU_749, but low values for the variable OTU_2018. Individuals belonging to this cluster exhibit these characteristic features. Besides, cluster 3 comprises individuals such as Var_Borbon and is identified by high values for variables like OTU_900, OTU_894, OTU_888, OTU_875, OTU_1116, OTU_857, OTU_852, OTU_765, OTU_727, and OTU_926. Finally, Cluster 4 is composed of individuals like Var_Castillo and is distinguished by high values for variables such as OTU_835, OTU_957, OTU_868, OTU_850, OTU_841, OTU_815, OTU_787, OTU_759, OTU_613, and OTU_496.

### Multivariate analysis of the fermentation bacterial microbiome about organoleptic properties

**Fig. 7:**
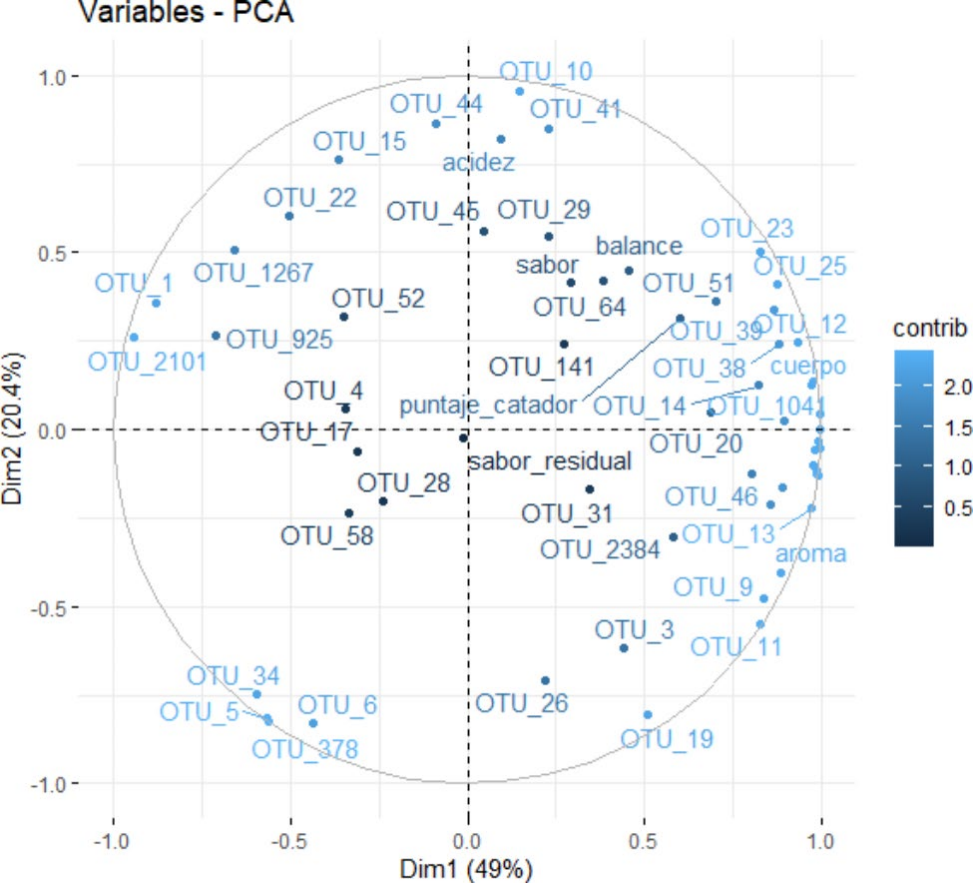
Multivariate analysis relating OTUs to organoleptic properties of coffee samples

A multivariate analysis, as depicted in Fig. 7, was performed to establish a relationship between OTUs and the organoleptic properties of coffee. The study encompassed five individuals and 59 variables and identified no outliers. The first two dimensions of the analysis accounted for 69.43% of the total variability in the dataset, signifying a strong association between OTUs and the sensory attributes of coffee. The analysis found that OTUs correlated strongly with specific organoleptic characteristics, including balance, flavor, residual flavor, body, aroma, and acidity. For example, it related the beverage balance to OTU_51 (non-laboratory cultivable bacteria of the species *Bacillus sp*), while it strongly related the flavor to OTU_64 and OTU_29 (uncategorized, non-cultivable bacteria). The residual flavor was closely related to OTUs 31 (*uncultured Solirubrobacter sp*) and 2384 (Pantoea sp), and the body was related to OTUs 104 (*uncultured Acidimicrobium sp*) and 12 (*uncultured Holophaga sp*). The aroma was related to OTU_13 (*Klebsiella pneumoniae*), and acidity was related to OTUs 46 (*uncultured Connexibacter*) and 44 (*Rhizobium sp*).

## Discussion

### Diversity and microbial richness in coffee beans

Various factors influence the diversity and richness of the rhizosphere microbiome during coffee bean fermentation. One of the factors is pH, which gradually decreases from a pH of 6.5 at T0 to pH of 4 at T24 [30], limiting the proliferation of microorganisms that are not adapted to a highly acidic environment. Bacterial structures change due to sugars; as the pH decreases, only bacteria tolerant to more acidic environments survive [31]. The diversity of the coffee bean microbiome is further impacted by the microorganisms’ requirement to penetrate the root tissues’ interior and move through vascular bundles to reach the bean [32]. Endophytic microorganisms, which establish interactions with plant cells, possess a competitive advantage in this process. Additionally, the lengthening of fermentation time reduces the availability of the substrate (sugar), which initiates resource competition among microbial communities [33].

Plant Growth-Promoting Rhizobacteria (PGPR) are beneficial bacteria that colonize the root surface, enhance plant growth and development, and may also influence the diversity of the rhizosphere microbiome. These PGPR can contribute to the fermentation process by producing enzymes that collapse complex organic compounds and release nutrients used by other microorganisms [34].

Another critical factor affecting the diversity and richness of the rhizosphere microbiome is plant defense compounds, including phytochemicals and allelochemicals. These compounds can have both inhibitory and stimulatory effects on the growth and activity of microorganisms. Their production depends on the plant species and the environmental conditions, and they can impact the microbial community structure and function in the rhizosphere [35].

In summary, multiple factors, such as pH, substrate availability, PGPR and plant defense compounds, and soil type and composition, can affect the diversity and richness of the rhizosphere microbiome during coffee bean fermentation. A comprehensive understanding of these factors can aid in optimizing the fermentation process and improving the quality of coffee beans.

### Influence of fermentation times and temperatures on bacterial community composition and microbial structure in coffee production

Regarding the characterization of microbial communities, the results of bacterial microbiome analysis revealed that increasing fermentation times and temperatures impacted the composition of bacterial communities, with the most growth observed in the *Proteobacteria* and *Firmicutes* phyla. It was also noted that the *Bacteroidetes* and *Actinobacteria* phyla disappeared with increasing fermentation time and temperature. The rhizosphere exhibited a greater diversity of phyla, including those typical of this environment (such as *Verrucomicrobia*, *Gemmatiomonadetes*, *Chloroflexi*, *Planctomycetes*, *Nitrospira*, and *Fusobacteria*), indicating that the microbial structure of the rhizosphere may be unique when compared to the fermentation production process. Additionally, the microbial structure of the rhizosphere for the Castillo-Tambo and Bourbon coffee varieties was found to differ in proportions. The dominant taxonomic orders in the fermentation production process were *Enterobacteriales*, *Rhodospirillales*, and *Lactobacillales*, while in the rhizosphere, the dominant orders were *Sphingomonadales*, *Sphingobacteriales*, *Rhizobiales*, *Burkholderiales*, *Actinomycetales*, *Verrucomicrobiales*, *Acidobacteriales*, *Solirubrobacteriales*, *Acidimicrobiales*, *Solibacterales*, *Gemmatimonadales*, *Nitrosomonadales*, *Desulfuromonadales*, *Ktedonobacteriales*, *Fusobacteriales*, *Syntrophobacterales*, and *Nitrospirales*. These findings provide insight into the influence of fermentation times and temperatures on the composition of bacterial communities and the unique microbial structure of the rhizosphere in coffee production.

The rhizosphere, which is the zone of soil surrounding the roots of a plant, plays a crucial role in the growth and development of coffee plants. The presence of microorganisms within the rhizosphere can impact the plant’s ability to resist pathogens, improve nutrient uptake, and optimize growth conditions. In particular, various species of *Proteobacteria*, *Firmicutes*, *Bacteroidetes*, and *Acidobacteria* have been found to participate in metabolic processes that impact the quality of coffee [36]. For instance, some species of *Proteobacteria*, including *Acetobacter*, produce enzymes that convert carbon dioxide into acetic acid during coffee fermentation, influencing the coffee’s flavor and acidity. Furthermore, some species of *Proteobacteria*, such as *Burkholderia* and *Pseudomonas*, produce volatile compounds that contribute to the aroma and flavor of coffee, and certain *Proteobacteria* species may play a role in protecting against pathogens and promoting plant growth [37].

*Firmicutes*, such as *Lactobacillus* and *Leuconostoc*, are known for their ability to ferment sugars, which can contribute to the production of volatile compounds and the acidity of coffee. Certain *Firmicutes* species, such as *Lactobacillus* and *Leuconostoc*, also produce diacetyl, a compound that impacts the flavor of the coffee [38].

Concerning the bacterial genera present in the fermented coffee samples, it can be deduced that a wide range of bacterial species are involved in the fermentation process, each of which plays an essential role at different stages. The results suggest a decrease in bacterial diversity as fermentation time progresses, and certain bacterial species seem to dominate the process more than others. Overall, the results suggest a high level of bacterial species diversity in all coffee samples, indicating a complex and intricate interaction between microorganisms and coffee during fermentation. The observed decrease in bacterial diversity over time suggests that only organisms capable of surviving under such conditions, such as the hardy *Tatumella* and *Pantoea*, prevail.

Specifically, the most abundant microbial genera include *Pantoea, Gluconobacter*, *Klebsiella*, and *Leuconostoc*. These genera are present in all samples, with a higher abundance in the more advanced fermentation samples. *Pantoea* and *Gluconobacter* are genera of lactic acid bacteria which are known to be involved in the production of lactic acid and acetic acid, which could contribute to the characteristic taste and aroma of coffee [39]. *Klebsiella*, conversely, is a genus of bacteria known to break down sugars and amino acids present in coffee, which could contribute to the release of aromatic compounds [40]. *Leuconostoc* is a genus of lactic acid bacteria known to produce lactic acid, which could contribute to the acidity of coffee [41].

Ultimately, it is essential to highlight the importance of the bacterial diversity in the samples, which suggests numerous microorganisms that can influence the coffee fermentation process. Therefore, it is imperative to properly characterize each of these microorganisms to identify those that have a determining influence on the properties of the final product and, in this way, improve its quality.

### Influence of coffee variety and fermentation on bacterial community diversity in coffee production

The analysis reveals that coffee variety influences beta diversity when separating samples in the plane. The biplot analysis identifies OTU_1, OTU_8, and OTU_3 as the main drivers of separation between rhizosphere and fermentation, with minor contributions from OTU_10, OTU_9, OTU_12, OTU_10, OTU_14, OTU_19, OTU_18, and OTU_20. Dendrogram (Fig. 6) analysis confirms this separation, observing four distinct groups of variables where original varieties are not related to samples with varying fermentation times. This suggests that bacterial communities change as fermentation progresses because of different biological interactions, such as nutrient competition or predation, which have been reported in other studies involving *Jeotgal* [42], *Glycine max* [43] and coffee [44].

When focusing on the two coffee varieties (Var_Cas and Var_Bor) without considering fermentation, it is observed that they are dissimilar to each other and grouped in different clades, which is reflected in elements such as height, leaf shape, and coffee produced from these species. This is because organisms interacting in their rhizosphere play a significant role in plant growth and its derivatives. In this line, Fig. 6 indicates that elevating the fermentation temperature heightens the metabolic rate of microorganisms, escalating their physiological activity and accelerating the fermentation processes [45].

A detailed analysis shows that OTU_1 is related to a group of bacteria that cannot be cultivated in the laboratory or have no taxonomic assignment, and its closest relative is *Uncultured Klebsiella sp*, while OTU_3 is related to *Pantoea agglomerans*. These bacterial groups are abundant in all fermentation samples and have been reported as microorganisms producing pectinase and organic acids related to improving the organoleptic characteristics of coffee beverages [46, 47]. OTU_8, OTU_10, OTU_9, OTU_12, OTU_10, OTU_14, OTU_19, OTU_18, and OTU_20 are related to non-culturable bacteria or those without taxonomic assignment and are mainly found in the rhizosphere samples of the two coffee varieties. These results suggest a wide diversity of microorganisms and open new research avenues to study their activity and role in the rhizosphere of coffee in the Castillo-Tambo and Bourbon varieties cultivated in the Popayán plateau and their potential relationship with organoleptic characteristics in coffee beverages. In [48], the importance of soil microorganisms in the cultivation of grapevines is described, highlighting how they influence fermentation and wine quality. Moreover, microbial biogeography’s relevance in wine production is denoted when considering how microbes interact with environmental conditions, thus driving wine quality, style, and denomination of origin.

### Exploring the role of non-cultivable microorganisms in improving the organoleptic characteristics of coffee

Most microorganisms investigated in this analysis were determined to be non-cultivable in laboratory conditions, thus emphasizing the necessity of further research to explore their potential impact on the organoleptic characteristics of coffee. Previous research on grapevine crops has demonstrated the presence and participation of particular microorganisms, such as *Rhizobium Pantone*, in the malolactic fermentation process, contributing to the formation of complex aromas in wine [49]. Furthermore, the quality and distinctiveness of wine from a specific geographic region have been linked to the composition of microorganisms present and their impact on wine fermentation attributes [48]. Future laboratory research should identify microorganisms that can enhance the organoleptic qualities of coffee cultivated in the Cauca department. This study should consider coffee’s microbial ecology and metabolome to produce a product with a potential origin denomination.

Apart from non-cultivable microorganisms, it is also crucial to consider the role of cultivable microorganisms in enhancing the organoleptic properties of coffee. Many yeast, bacterial, and fungal species have been detected in coffee beans’ wet processing fermentation process, which may impact the end product’s taste, aroma, and other sensory attributes. The composition and quantity of microorganisms present during fermentation can vary depending on coffee beans’ origin and processing methods, environmental conditions, and cultivation management practices [15].

Overall, it is evident that microorganisms play a significant role in shaping the organoleptic characteristics of coffee. Therefore, further research is essential to explain the specific contributions of both cultivable and non-cultivable microorganisms to optimize the sensory qualities of this beloved beverage.

Finally, the present study has limitations worth noting. First, the obtained results are susceptible to the influence of agro-climatic factors that the coffee plant was exposed to during its life cycle. Second, the data were collected during a specific timeframe, which may reflect something other than the coffee plant’s overall growth and development. It is crucial to acknowledge that the study’s findings may vary significantly depending on the coffee plant’s age, with this study only using samples from three-year-old coffee plants. Therefore, it is imperative to recognize that it cannot extend the obtained results to other age groups of coffee plants or diverse coffee-growing regions within Colombia with distinct soil and climatic conditions.

## Conclusion

In this research, the microbial community of the soil of coffee trees of the Bourbon and Castillo varieties grown on the Popayán-Cauca plateau was characterized using DNA sequencing. The microorganisms found in the rhizosphere were then compared to the organoleptic properties of the coffee, as determined by cupping according to ISO 17.025. The results suggest that each variety of coffee has a distinct microbial profile, which may be related to the plants’ physiological, nutritional, and sanitary needs. Furthermore, the investigation revealed a rich microbial diversity in the soil and during fermentation, with several microorganisms belonging to bacterial taxa that are not amenable to laboratory cultivation. This suggests the potential for identifying microorganisms of agronomic interest and further understanding their role in the life cycle of coffee plants.

While investigating the fermentation process of coffee beans on the Popayán plateau, it was observed that the phyla *Proteobacteria* and *Firmicutes* were the dominant bacterial taxa, independent of the fermentation temperature performed. During this process, examination of the microbial community revealed a high level of diversity and richness in microorganisms colonizing endocarp surfaces. Additionally, it was noted that the bacterial community underwent structural changes because of sampling time and temperature variations. In this line, further investigations are required to fully comprehend the significance and function of these microorganisms in the catabolic process of coffee fermentation, employing similar methodologies to those utilized in the wine production industry.

Finally, the research demonstrated a strong relationship between specific rhizospheric microorganisms and coffee’s organoleptic properties, such as flavor, acidity, balance, and residual flavor. Therefore, future work could focus on improving these characteristics by manipulating specific microbial traits to optimize the organoleptic properties of the coffee beverage. Many of the microorganisms that influence the physical attributes of coffee are not culturable, indicating that techniques such as metagenomics or metabolomics may be necessary for a comprehensive analysis of their role in coffee fermentation processes.

## Materials and methods

### Location

It conducted the experiments at two locations, namely the Corporación Universitaria Comfacauca - Unicomfacauca, in Popayán and the hacienda Los Naranjos of La Venta in the Cajibio municipality, owned by the Parque Tecnológico de Innovación del Café (TECNICAFE). The geographical coordinates of the experimental sites are 2°35’11.6” N and 76°33’11.2” W, in the Cauca department of Colombia. It grew the crop approximately 1862 m above sea level, while the average temperature ranges between 12 and 23 °C [50].

### Plant material

The present investigation employed plant materials from Hacienda Los Naranjos in Cajibio. Specifically, two distinct varieties of the coffee plant, namely Bourbon and Castillo Tambo, were chosen based on their unique characteristics and growth habits. The Bourbon variety exhibits a tall growth habit and moderate yield, with the potential to produce high-quality coffee at high altitudes [51]. Nonetheless, it is no longer cultivated in the region because of its susceptibility to rust attacks. The Castillo Tambo variety is a hybrid of Caturra and Timor Hybrid varieties, which provide rust resistance, high productivity, excellent beverage quality, and adaptability to diverse coffee ecotypes [52]. Samples were collected from two-year-old trees, and it analyzed both the roots and coffee beans to evaluate the bacterial microbiome during the fermentation process and in the rhizosphere.

### Fermentation trials and cup profiles

At TECNICAFE, 10 kg of cherry coffee at different stages of commercial maturity were harvested and processed. It transported the beans in refrigerated compartments to Supracafé’s processing plant [53], where they underwent a semi-washing process using a pulper to produce Baba coffee [54]. The first processing stage included removing the exocarp through the pulping process and it placed the samples at 4 °C to prevent fermentation. Finally, the samples were transported to the Corporación Universitaria Comfacauca - Unicomfacauca for further experimentation.

The experimental design involved subjecting the coffee beans to fermentation under two different temperature conditions: ambient (19.5 °C) and warm (24 °C), to simulate the environmental conditions of the Popayán plateau [55], where the analyzed samples were obtained. Samples were taken and subjected to aerobic fermentation, extracting samples in triplicate at 0, 12, and 24 h, labeled as T0 (zero fermentation time), T12A (sampling at 12 h at ambient temperature), T24A (sampling at 24 h of fermentation at ambient temperature), T12C and T24C (sampling at 12 and 24 h at a hot temperature, respectively). Subsequently, the samples were stored at -20 °C for DNA extraction. In addition, the degree Brix and pH were quantified.

Upon completion of the fermentation process, it washed the samples with abundant water to eliminate mucilage and dried them to achieve 11% grain moisture. The cup profile of the samples was determined by sizing, threshing, and sieving the beans in 14 mesh, followed by the analysis of physical defects of the coffee based on the recommendation of the Federación Nacional de Cafeteros [1]. Two expert and certified tasters evaluated the quality of the coffee following the method established by the Specialty Coffee Association of America (SCAA) [56]. The qualification values were calculated according to the ISO 17.025 procedure of Almacafé. In this line, It is crucial to specify that the organoleptic properties examined in this study encompassed the ultimate evaluations of aroma, flavor, acidity, body, uniformity, balance, sweetness, and aftertaste.

### Coffee rhizosphere assays

This investigation collected soil samples from the rhizosphere of two coffee varieties, Bourbon and Castillo-Tambo, in Cauca, Colombia. It carefully obtained the rhizosphere soil by removing the top 5 cm of soil surrounding the coffee plant base while minimizing damage to the root system. This technique is widely used in rhizosphere studies and has been described in the scientific literature [57].

To differentiate between rhizosphere and bulk soil, it collected rhizosphere samples in duplicate from 15 plants per replicate, whereas it collected bulk soil samples from the exact location but at least 5 millimeters away from the coffee plant base [58]. Then, to ensure the rhizosphere’s integrity, the top layer of soil adhering to the roots under analysis was removed using a spatula. This method is commonly employed in studies that distinguish rhizosphere soil from bulk soil [59, 60].

Subsequently, the soil samples were transported to the biotechnology laboratory of Corporación Universitaria Comfacauca-Unicomfacauca, where rhizosphere soil from Bourbon (Var_Bor) and Castillo-Tambo (Var_cas) coffee varieties were extracted in duplicate from the coffee roots. The extracted samples were stored at -20 °C for further analysis.

### Extraction of DNA from coffee mucilage and rhizosphere

It maintained the collected samples at -20 °C until further use. Upon thawing, it immediately processed the samples at 20 °C for DNA extraction from the mucilage and rhizosphere. Following the manufacturer’s recommended procedure, a commercial DNeasy PowerSoil kit from QIAGEN [61], following the manufacturer’s recommended procedure. It diluted the extracted DNA with ultrapure water free of DNases and RNases and then stored at -20 °C for further use.

The quality of the extracted DNA was analyzed on a 0.8% (w/v) agarose gel and quantified with the NanoDrop 2000 spectrophotometer (Thermo Scientific, Waltham, MA, USA) [59]. The bacterial community in the rhizosphere of the Castillo and Bourbon varieties was amplified by Polymerase Chain Reaction (PCR) of the V4 region of the 16S rRNA genes. For this, forward illCUs515F 5’-GTGYCAGCMGCCGCGGTAA [62] and reverse new806RB 5’-GGACTACNVGGGTWTCTAAT [63] primers were used at a concentration of 20ng/µL. The reaction mixture had a 100 µL, containing 200 µM of dNTPs, 2.5 mM MgCl2, 0.5% DMSO, 1.25 U of Go Taq Polymerase, and 20 ng of metagenomic DNA. PCR was performed on a GenePro BIOER thermal cycler, following the cycling conditions of initial denaturation at 95 °C for 10 min, followed by 28 cycles of denaturation at 95 °C for 45 s, hybridization at 53 °C for 45 s and elongation at 72 °C for 5 min.

Amplification products were analyzed by electrophoresis on 1% (w/v) agarose gels and stained with ethidium bromide. Electrophoresis was performed by applying 100 volts for 15 min on a MiniRun GE-100, and the gels were visualized on a transilluminator. The amplified fragments were purified with the commercial Accuprep kit (BIONEER) and eluted in a final volume of 30 µl. Samples were sent to Molecular Research LP (MR DNA) laboratory in the USA for sequencing by Illumina MiSeq technology [60].

### Statistical analysis of the samples

The sequencing data was analyzed using the Mothur platform tools [64]. Reads that did not meet specific criteria were removed, including reads with a size less than 100 base pairs (bp), a sequence difference (mismatch) in the barcode, and reads not aligned with the SILVA database [65] using the UCLUST method [66], Furthermore, the UCHIME method [67]. They removed chimeras from the sequence data. OTUs were formed by clustering sequences with a 95% similarity threshold, and it eliminated OTUs with less than three sequences to minimize sequencing errors, following similar procedures as in previous studies [68, 69].

The estimation of microbial community richness was determined using rarefaction curves with the resampling method without replacement by the Mothur settings. To estimate diversity indexes, such as Chao-1 [70] and Shannon richness (H’) [71] of the microbial community, the program Past [50] was employed. ANOVA was used to analyze the obtained data, and the InfoStat software [72], was used to perform Tukey’s test to compare means with a 5% significance level (P ≤ 0.05).

Likewise, a multivariate analysis comprising PCA [73] and cluster analysis [74], were performed to identify relationships between variables and individuals. To investigate the association between genetic samples and fermentation times, OTUs with over ten sequences were selected, resulting in 1265 individuals. To explore the correlation between OTUs and organoleptic properties, it reduced the number of individuals to 60 by eliminating those with fewer than 500 genetic sequences.

## Data Availability

The datasets utilized in this study have been made available in The Knowledge Network for Biocomplexity (KNB) repository, under the identifier 10.5063/F1HM56WB, for further research and analysis.
